# Lost in Time and Space: States of High Arousal Disrupt Implicit Acquisition of Spatial and Sequential Context Information

**DOI:** 10.3389/fnbeh.2017.00206

**Published:** 2017-11-09

**Authors:** Thomas Maran, Pierre Sachse, Markus Martini, Barbara Weber, Jakob Pinggera, Stefan Zuggal, Marco Furtner

**Affiliations:** ^1^Department of Psychology, University of Innsbruck, Innsbruck, Austria; ^2^Department of Educational Sciences and Research, Alps-Adria University of Klagenfurt, Klagenfurt, Austria; ^3^Department of Applied Mathematics and Computer Science, Technical University of Denmark, Kongens Lyngby, Denmark; ^4^Department of Computer Science, University of Innsbruck, Innsbruck, Austria; ^5^Department of Entrepreneurship, University of Liechtenstein, Vaduz, Liechtenstein

**Keywords:** arousal, stress, context processing, associative learning, spatial learning, multiple memory systems

## Abstract

Biased cognition during high arousal states is a relevant phenomenon in a variety of topics: from the development of post-traumatic stress disorders or stress-triggered addictive behaviors to forensic considerations regarding crimes of passion. Recent evidence indicates that arousal modulates the engagement of a hippocampus-based “cognitive” system in favor of a striatum-based “habit” system in learning and memory, promoting a switch from flexible, contextualized to more rigid, reflexive responses. Existing findings appear inconsistent, therefore it is unclear whether and which type of context processing is disrupted by enhanced arousal. In this behavioral study, we investigated such arousal-triggered cognitive-state shifts in human subjects. We validated an arousal induction procedure (three experimental conditions: violent scene, erotic scene, neutral control scene) using pupillometry (Preliminary Experiment, *n* = 13) and randomly administered this method to healthy young adults to examine whether high arousal states affect performance in two core domains of contextual processing, the acquisition of spatial (spatial discrimination paradigm; Experiment 1, *n* = 66) and sequence information (learned irrelevance paradigm; Experiment 2, *n* = 84). In both paradigms, spatial location and sequences were encoded incidentally and both displacements when retrieving spatial position as well as the predictability of the target by a cue in sequence learning changed stepwise. Results showed that both implicit spatial and sequence learning were disrupted during high arousal states, regardless of valence. Compared to the control group, participants in the arousal conditions showed impaired discrimination of spatial positions and abolished learning of associative sequences. Furthermore, Bayesian analyses revealed evidence against the null models. In line with recent models of stress effects on cognition, both experiments provide evidence for decreased engagement of flexible, cognitive systems supporting encoding of context information in active cognition during acute arousal, promoting reduced sensitivity for contextual details. We argue that arousal fosters cognitive adaptation towards less demanding, more present-oriented information processing, which prioritizes a current behavioral response set at the cost of contextual cues. This transient state of behavioral perseverance might reduce reliance on context information in unpredictable environments and thus represent an adaptive response in certain situations.

## Introduction

Cognitive adaptations during high arousal states play an important role in models of development of psychopathology, e.g., post-traumatic stress disorder or phobias (Acheson et al., 2012; Pitman et al., 2012; de Quervain et al., 2017), shooting decisions during police actions (Nieuwenhuys et al., 2015), implementation of military operations on the battlefield (Lieberman et al., 2016) and forensic considerations in the face of crimes committed in rage (Brookman, 2015). Thus, examining cognitive functioning in humans *during* extreme arousal states has important implications on basic and applied research. Beyond everyday fluctuations of general arousal (Berridge, 2008; Carter et al., 2010; de Lecea et al., 2012; Nielsen and Mather, 2015; Nielsen et al., 2015), extreme increases occur in response to challenging situations (Berridge and Waterhouse, 2003), such as panic or sexual excitement (Calderon et al., 2016), and promote fundamental changes in cognition in response to immediate environmental demands (Shields et al., 2016a). The current study complements existing research on arousal effects on cognition, examining whether states high in arousal alter sensitivity for spatial relations or sequential order, two core aspects of episodic memory formation.

Temporal-spatial context of a past event represents an immanent part of episodic memory formation (Tulving, 1984). Encoding and/or retrieval of both types of context information have been shown to strongly rely on a single hippocampus- and prefrontal cortex-dependent memory system (Rajah et al., 2010a,b, 2011; Kraus et al., 2013; Cabral et al., 2014). This network is supposed to support the construction of a schematic model of a situation based on contextual information (Bar, 2004; Ranganath and Ritchey, 2012), such as spatial and temporal relationships, therefore it is required to form contextualized, detailed representations of events and for allocentric spatial navigation (Squire et al., 2004; Howard and Eichenbaum, 2013; Pezzulo et al., 2014; Davachi and DuBrow, 2015). However, processing of spatial and temporal details is prone to interference by acute stress. At least for long-term memory formation, it has been shown that events encoded immediately after stressful encounters are embedded less in contextual details and lack precision (Schwabe et al., 2009). In addition, encoding during arousal leads to more gist-like memories at the cost of peripheral details (Kensinger et al., 2007). This narrowed focus on the essential core of experiences lacking contextual embeddedness and depth of detail is reflected by an increased rate of false alarms (Payne et al., 2002) and is directly related to autonomic arousal (Qin et al., 2012).

An explanation for these findings is offered by a recent account that builds on evidence showing that acute stress leads to an upregulation of the salience network (Hermans et al., 2011, 2014), which then biases competition of learning and memory between multiple underlying systems (Poldrack and Packard, 2003; Mizumori et al., 2004; Squire and Dede, 2015). Stress produces a shift toward the use of “habit” memory by impairing hippocampus- and probably prefrontal cortex-dependent “cognitive” memory (Arnsten et al., 2012; Packard and Goodman, 2012; Schwabe and Wolf, 2013; Schwabe, 2013, 2016; Gagnon and Wagner, 2016). Existing evidence fits in well with this notion, showing that stressful encounters decrease hippocampal activation (Pruessner et al., 2008; Henckens et al., 2009; Cousijn et al., 2012; Schwabe and Wolf, 2012) and thereby strengthen the dorsal striatum-dependent system (Poldrack and Packard, 2003; Vogel et al., 2015a, 2017), which then supports incremental strengthening of stimulus-response associations (Packard and Knowlton, 2002; Devan et al., 2011).

With a focus on cognitive functioning, stress hijacks active cognition by impairing working memory (Schoofs et al., 2008, 2009; Luethi et al., 2009; Qin et al., 2009), cognitive flexibility (Plessow et al., 2011, 2012; Shields et al., 2016b) and cognitive inhibition (Mahoney et al., 2007; Sänger et al., 2014), but simultaneously enhances behavioral control in terms of response inhibition (Schwabe et al., 2013; Weinbach et al., 2015). At the same time, increases in arousal lead to reduction of the range of cue use by narrowing attention to prioritized cues at the expense of surrounding information (Harmon-Jones et al., 2013; Sakaki et al., 2014; Weinbach and Henik, 2014; Maran et al., 2017). In addition, working memory performance under stress is characterized by a higher rate of false alarms, indicating less specific representations and more liberal responding (Duncko et al., 2009). Schwabe and Wolf (2013) pointedly termed this bias of the relative use of multiple memory systems a shift “from thinking to doing”.

Altogether, these findings provide strong evidence for dynamic adjustment of ongoing information processing depending on arousal state, suggesting a switch from contextual “cognitive” to rigid “habit” strategies underlying active cognition, leading to reduced reliance on contextual information. The current study focuses on this suggested consequence of high arousal states on cognitive performance, meaning impaired ability to use contextual cues to inform actual responses. The first aim of this study was to examine how high arousal states affect two core aspects of context processing, the acquisition of spatial (Experiment 1) and sequential (Experiment 2) context information. More specifically, we focused on whether aroused subjects *implicitly* acquire contextual details by assessing how context information affects performance depending on different arousal states. Although evidence strongly indicates that arousal, regardless of its motivational direction, drives adaptations in cognition (Mather and Sutherland, 2011; Harmon-Jones et al., 2013; Maran et al., 2015), previous work on this topic focused solely on the effect of aversive stressors (e.g., fear). Thus, the second aim of this study was to investigate whether both aversive and appetitive arousal states (i.e., sexual excitement) lead to similar alterations of context acquisition and thereby further support the notion that the tested cognitive adaptations are mainly being driven by variations in arousal rather than emotional valence.

We implemented two experimental designs to measure the implicit acquisition of spatial and sequential context, each with arousal state varying as between-subject condition. Our first prediction proposes that states high in arousal disrupt implicit acquisition of context information, more specifically the use of both spatial and sequential context of ongoing events. Since the moment-to-moment processing of contextual details facilitates task execution in both paradigms described below, impaired performance would support this first hypothesis. Second, we expect that increases in arousal exert their effects regardless of its motivational direction, thus exposure to both aversively and appetitively arousing events should result in the same performance decrements. Evidence showing any differences in arousal-induced performance decrements between the aversive and appetitive states would support this second hypothesis.

Our predictions were tested in two behavioral experiments using established experimental paradigms, assessing implicit acquisition of spatial relations (spatial discrimination paradigm; Marshall et al., 2016; Experiment 1) and predictive sequences (learned irrelevance paradigm; Orosz et al., 2008, 2011; Experiment 2). The elicitation procedure comprised three cinematographic fragments: a social conversation, a violent encounter and sexual intercourse. To ensure the effectiveness of the arousal elicitation method, we conducted a preliminary study to evaluate the procedure by measuring pupillary responses. We implemented the arousal elicitation method between practice and testing, therefore the arousing encounter can be considered as part of the task context (Joëls et al., 2006; Diamond et al., 2007). Since effects of arousal on cognition are strongly time dependent (Joëls and Baram, 2009; Joëls et al., 2011; Henckens et al., 2012), it is noteworthy that we focused on immediate cognitive adaptations after exposure to an arousing event. Thus, both tasks took less than 20 min to perform, well before cortisol secretion peaks in response to an arousing encounter (after about 25 min, Schwabe et al., 2008).

In the following, we first present the Preliminary Experiment which aimed to validate the general arousal elicitation procedure by analyzing tonic pupillary changes and self-report mood states (Preliminary Experiment: Validation of the general arousal elicitation procedure). Second, in Experiment 1 we examined whether states high in arousal affect implicit acquisition of spatial context (Experiment 1: Arousal and spatial context processing) and third, Experiment 2 explored how alterations in arousal impact acquisition of sequence information (Experiment 2: Arousal and sequence acquisition).

## Preliminary Experiment: Validation of The General Arousal Elicitation Procedure

In the following experiment, cinematographic material was validated by analyzing tonic pupillary changes with regard to their ability to induce different states of arousal. Three scenes from existing feature films were chosen, the first scene showing a casual conversation during shopping (control condition), the second scene a highly aversive, violent homicide (aversive arousal) and the last clip romantic, explicitly sexual intercourse (appetitive arousal). Although we investigated effects of arousal referring to these distinct experimental conditions, it should be noted that arousal is a continuous neurobiological function (Pfaff and Banavar, 2007).

The use of emotional clips in order to induce arousal states represents an established and efficient method (e.g., Gabert-Quillen et al., 2015; Samson et al., 2015; Gilman et al., 2017). Since the chosen scenes depict realistic events, they match extremely arousing situations in real life which can be considered as unpredictable and uncontrollable (i.e., involvement in violent encounters or sexual interactions).

Validation of the method used was ensured by comparison of tonic pupil response immediately after stimulus exposure. Pupillary dynamics has been shown to be a reliable, non-invasive indicator of arousal (Bradley et al., 2008), mediating neuromodulatory actions (Hou et al., 2005) and corresponding locus coeruleus recruitment (Rajkowski et al., 1994; Murphy et al., 2014). Furthermore, recent evidence shows that locus coeruleus activity anticipates changes in pupil diameter (Joshi et al., 2016). Thus, since we expect arousal to be the driving force behind the hypothesized disruption of implicit acquisition of context information, capturing differential tonic pupil responses between conditions represents a precise physiological marker to validate the arousal elicitation procedure used for both of the following experimental designs.

To be considered effective to induce a state of enhanced arousal, the scene presenting violent and sexual interactions should induce a larger pupil dilatation compared to the control clip showing an everyday life scene. In addition, since the material should induce arousal states of different valence, i.e., aversive and appetitive, self-reported mood states should reflect specific alterations in negative and positive affect, respectively.

### Materials and Methods

#### Design and Procedure

In a within-subject design, participants were presented with three different cinematographic scenes (control, erotica, violence). The subjects saw an initial fixation cross for the duration of 7 s, followed by a 60 s clip and another fixation cross of 90 s. Each participant watched all three clips between 10–12 am on consecutive days, approximately 24 h apart. We presented one clip a day in a randomized order. Pupil diameter was sampled throughout the procedure by a Tobii TX 300 eye-tracker. Subjects were sat 30 cm away from the eye-tracker.

To check the effects on positive and negative affectivity, we registered participants’ affect at the beginning and the end of the experimental procedure by using the “Positive and Negative Affect Schedule” (PANAS; Watson et al., 1988; German translation by Krohne et al., 1996, five-point Likert scale). This scale allows capturing current mood states by an evaluation of a series of words which describe various feelings.

#### Participants

Thirteen young adult participants (6 females, 7 males; *M*_age_ = 23.77 years, *SD* = 2.89; age range: 20–30 years) were healthy volunteers recruited from the University of Innsbruck and received research credits for participation in the experiment. All had normal or corrected-to-normal visual acuity. None of the participants indicated suffering from a diagnosed psychiatric disease or having first-degree relatives who did, being under the influence of psychoactive substances or psychopharmacologic treatment, or having suffered severe head injuries in the course of their lives (self-report). In addition, participants had no history of being exposed to a severe traumatic event, had frequently watched violent movies or played violent video games. This study was carried out in accordance with the recommendations of the guidelines of the Ethics Committee of the Department of Psychology, University of Innsbruck, with written informed consent from all subjects. All subjects gave written informed consent in accordance with the Declaration of Helsinki. The protocol was approved by the Ethics Committee of the Department of Psychology, University of Innsbruck.

#### Arousal Elicitation Procedure

Three short fragments from existing feature films were used to induce altered states of arousal. In doing so, we intended to experimentally induce three sustained states with the longest possible duration during completion of the tasks described below: (a) a neutral, low arousal state serving as control condition; (b) an aversive, high arousal state; and (c) an appetitive, high arousal state. The selected fragment for induction of valence after highly aversive arousal showed a distressing scene of violence (an aggressive and violent encounter between men during which one gets killed by a fire extinguisher) and that for induction of highly appetitive arousal showed an explicitly sexual scene (a man and woman during sexual intercourse, including close-up images of genitalia), whereas a social interaction (shopping scene featuring two women) was presented as control condition.

All selected fragments had matched audiovisual characteristics. The first and last scenes have successfully been used in previous studies investigating states of stress (e.g., Hermans et al., 2011). Providing both an appetitive and aversive state of high arousal allows for experimentally ruling out specific alterations due to the valence of the scenes and therefore the motivational direction of a corresponding state (Mather and Sutherland, 2011; Harmon-Jones et al., 2013).

The cinematographic material was approved by the Austrian Commission for Media for Youth (JMK) for viewers above 16 years and participants have previously been informed that the scenes they were about to watch might contain offensive or distressing content. Subjects could end their participation in the experiment at any time if desired.

#### Pupil-Diameter Measurements

Pupil diameter was sampled at 300 Hz and recorded throughout the task using an infrared video eye-tracker (Tobii TX 300). Pupil-diameter measurements were processed using a newly developed open source tool, Cheetah Experimental Platform Web, 2.0 (CEP–Web; Zugal et al., 2017), which allows performing the following evaluation stages. First, we substituted any missing values from one pupil by the values determined for the respective other pupil. Second, all values that differed more than three standard deviations from the mean value were considered outliers and therefore removed. Third, the blink detection filter implemented in CEP–Web which, based on a heuristic of missing values and gaze position, detects and clips out blinks 200 ms section before and after each identified blink, (Pedrotti et al., 2011). Next, a filter for linearly interpolating missing data by linear interpolation of values measured just before and after each blink provided by CEP-Web was applied. Finally, based on the continuous pupil measurements, data were low-pass filtered using a third order low pass Butterworth with a cutoff frequency of 4 Hz. Even though a low pass Butterworth filter can be used for processing measurement artifacts, its application introduces a phase response to the filtered signal towards the past. In order to compensate for this phase response, CEP-Web calculates the expected phase response and automatically reshifts the processed signal so that the phase response is equalized.

Relative pupil dilatation was calculated using the mean pupil dilatation 5000 ms before the scene as baseline. Since we were not interested in the pupillary response during the scenes, we focused on mean relative pupillary dilatation during the duration of the second fixation cross as an index of sustained alteration in tonic arousal. Measurements within the first 2000 ms were removed, since they reflected the initial pupillary adaptation to the low level differences between the cinematographic material and the following fixation cross.

#### Data Analysis

To examine the effects of different scenes on tonic pupil dilatation as well as self-reported mood and arousal, an ANOVA for independent measures was applied to the preprocessed mean pupil-diameter measurements with scene type (neutral, violence, erotica) as between-subject variable.

Sphericity was tested using Mauchly’s test and in case of deviance from sphericity, Type I error was controlled by adjusting the degrees of freedom using the Greenhouse-Geisser correction. All reported *p* values are two-tailed. Alpha levels were set at 0.05. In addition, we applied Bayesian inferential procedures for each hypothesis testing, which allows quantifying the relative strength of evidence for one hypothesis compared to the other. Data were analyzed using SPSS (Version 24) and JASP (Version 0.8; JASP Team 2016).

### Results

Results from all participants were subject to data analysis. Effect sizes are reported by partial eta squared $\eta_{\text{Part}}^{2}$ (0.01 = small; 0.06 = medium; 0.14 = large) for analyses of variance.

First, we applied repeated measures ANOVAs on pupil dilatations during presentation of the second fixation cross immediately after the arousal-inducing material. Two dependent variables were defined, pupillary response relative to a 5 s baseline before observation of the scenes and absolute pupil dilatation in pixels during the second fixation interval (Table 1). Considering the small sample size, we additionally applied non-parametric analyses.

**Table 1:** Effects of different conditions of the arousal elicitation method on relative and absolute tonic pupil dilatation as well as self reported mood.

	Arousal state					
	Control		Violence		Erotica	
Lure-Type	*M*	*(SE)*	*M*	*(SE)*	*M*	*(SE)*
Tonic pupil size_Rel._	0.92	(0.01)	0.96	(0.01)	0.96	(0.01)
Tonic pupil size_Abs._	2.94	(0.03)	3.14	(0.03)	3.05	(0.02)
Negative affectivity	−0.29	(0.16)	−0.28	(0.11)	0.47	(0.14)
Positive affectivity	0.12	(0.11)	0.87	(0.15)	0.17	(0.12)

*Standard errors in parentheses (standard errors were corrected for repeated measures)*.

Statistical testing on relative pupil sizes indicated a strong main effect for the arousal group, *F*_(2,24)_ = 5.07, *MSE* = 0.001 *p* = 0.015, $\eta_{\text{Part}}^{2}$ = 0.30, *BF*_10_ = 3.98. Mauchly’s test showed that the assumption of sphericity had been fulfilled for the within-subject variable arousal condition, $\chi_{\left(2\right)}^{2}$ = 0.57, *p* = 0.168. Whereas pupillary response did not differ between high arousal groups, Δ = 0.01, *SE* = 0.02, Bonferroni-adjusted *p* = 1, compared to the neutral group, relative pupil size was higher for both the aversive group with a difference of 0.04 (*SE* = 0.01), Bonferroni-adjusted *p* = 0.017, and in the appetitive group with a difference of 0.03 (*SE* = 0.01), Bonferroni-adjusted *p* = 0.033. Likewise, absolute pupil response differed across arousal conditions, *F*_(2,24)_ = 10.63, *MSE* = 0.012 *p* < 0.001, $\eta_{\text{Part}}^{2}$ = 0.47, *BF*_10_ = 51.29. Mauchly’s test confirmed that the assumption of sphericity had been fulfilled for the within-subject variable arousal condition, $\chi_{\left(2\right)}^{2}$ = 0.70, *p* = 0.705. Again, the aversive and appetitive conditions did not differ regarding absolute pupil size during the fixation interval after the arousing material, Δ = 0.08, *SE* = 0.04, Bonferroni-adjusted *p* = 0.191. By contrast, participants’ pupil dilatation was larger after the violent clip compared to the control clip with a difference of 0.20 (*SE* = 0.05), Bonferroni-adjusted *p* = 0.004, and even more enlarged after exposure to the appetitive compared to the neutral material with a difference of 0.11 (*SE* = 0.04), Bonferroni-adjusted *p* = 0.041.

In addition, Friedman’s tests overall confirmed these effects showing alterations in relative, $\chi_{\left(2\right)}^{2}$ = 6.00, *p* = 0.050, and absolute tonic pupil responses, $\chi_{\left(2\right)}^{2}$ = 12.91, *p* = 0.002, after application of the arousal induction procedure. These results support the validity of the arousal elicitation method, showing that both cinematographic fragments, the violent and explicitly sexual scenes, induced a tonically enlarged pupil dilatation, which indicates a state of increased arousal.

Second, in order to assess the effects of the arousal induction method on current subjective mood, repeated measures ANOVAs were applied on changes in mood state, calculated by subtracting mood state values before and after participants underwent the procedure. For negative affectivity, Mauchly’s test indicated that the assumption of sphericity had been fulfilled for the within-subject arousal condition, $\chi_{\left(2\right)}^{2}$ = 1.29, *p* = 0.525. Results revealed a strong effect of arousal conditions on self reported negative affect state, *F*_(2,24)_ = 7.22, *MSE* = 0.319, *p* = 0.004, $\eta_{\text{Part}}^{2}$ = 0.38, *BF*_10_ = 25.71. Participants in the aversive arousal condition reported higher negative mood compared to those in the low arousal control condition with a difference of 0.75 (*SE* = 0.25), Bonferroni-adjusted *p* = 0.020, as well as compared to participants in the appetitive arousal condition with a difference of 0.70 (*SE* = 0.23), Bonferroni-adjusted *p* = 0.045. By contrast, there was no difference between the control group and the appetitive arousal group, Δ = 0.05, *SE* = 0.18, Bonferroni-adjusted *p* = 1.

Regarding positive affectivity, again we found a strong main effect for arousal group, *F*_(2,24)_ = 6.43, *MSE* = 0.387 *p* = 0.006, $\eta_{\text{Part}}^{2}$ = 0.35, *BF*_10_ = 16.84. Mauchly’s test indicated that the assumption of sphericity had been fulfilled for the within-subject arousal condition, $\chi_{\left(2\right)}^{2}$ = 1.99, *p* = 0.371. Specifically the appetitive arousal group reported higher positive mood compared to both the aversive arousal group with a difference of 0.75 (*SE* = 0.20), Bonferroni-adjusted *p* = 0.008, and the low arousal control group, with a slightly significant difference of 0.76 (*SE* = 0.29), Bonferroni-adjusted *p* = 0.062. On the other hand, the latter two groups did not differ with regard to self-reported positive affect, Δ = 0.01, *SE* = 0.24, Bonferroni-adjusted *p* = 1.

Friedman’s tests confirmed these effects, showing alterations in mood state between repeated measures for both negative, $\chi_{\left(2\right)}^{2}$ = 10.80, *p* = 0.005, and positive affect, $\chi_{\left(2\right)}^{2}$ = 12.04, *p* = 0.002.

Altogether, results of the self-report data confirm successful induction of the targeted differences in valence, showing that arousal induction conditions induce states with opposite motivational direction (aversive and appetitive).

Our results provide strong evidence that both of the selected cinematographic fragments, which depicted violence and erotica, presented an effective measure to elicit a state of increased arousal and different valence. Participants showed increased pupil sizes following exposure to the high arousal scenes compared to the control clip. The uniform tonic change of pupil dilatation in response to the arousal-inducing material suggests that the material used meets the criteria to experimentally create a situation of sufficient strength (Lissek et al., 2006), thus being able to effectively evoke the targeted normative cognitive adaptations as aimed for in the following experiments.

## Experiment 1: Arousal and Spatial Context Processing

Spatial context represents a crucial source of information in everyday life as it informs spatial navigation in terms of way-finding (Wiener et al., 2004). Notably, both working memory for spatial context as well as memory encoding and retrieval thereof have been shown to be a hallmark of hippocampal function (Rajah et al., 2010a,b; Spellman et al., 2015; Esfahani-Bayerl et al., 2016). Yet, the engagement of hippocampal-centered processing in learning and memory has been shown to be impaired by increased arousal (Packard and Goodman, 2012; Schwabe and Wolf, 2013). In fact, acute stress is associated with an impairment of spatial working memory in rodents and monkeys (Gamo et al., 2015), healthy humans (Moriarty et al., 2014; Olver et al., 2015) and psychiatric populations (Smith and Lenzenweger, 2013), as well as impaired spatial learning in rodents (Akirav et al., 2001, 2004; Herrero et al., 2006) and monkeys (Arnsten and Goldman-Rakic, 1998). In humans, exposure to stress strengthens reliance on egocentric, route-based strategies at the cost of allocentric, cognitive map-based context information (van Gerven et al., 2016; Brunyé et al., 2017). Moreover, further research showed that under acute stress, spatial navigation is supported less by a hippocampus-dependent strategy, which maps flexible spatial relations using multiple cues (Schwabe et al., 2007; Vogel et al., 2017).

To assess whether states high in arousal affect implicit acquisition of spatial context, we applied the spatial mnemonic discrimination paradigm (Reagh et al., 2014). After participants underwent the arousal induction procedure, they had to respond to a sequence of objects, which were presented at different spatial locations. In a subsequent surprise recall, participants viewed the same objects at either the same or stepwise vertically or horizontally displaced locations and had to decide whether the object had remained in the same location or whether it had been moved. Recently, this paradigm has been applied successfully to study early forms of hippocampal impairment in elderly humans (Reagh et al., 2014) and the impact of experienced life-time stress on age-related hippocampal function (Marshall et al., 2016). In addition, spatial discrimination ability in this spatial task was related to performance in the Rey Auditory Verbal Learning Test, a neuropsychological test of hippocampus related, declarative memory (Reagh et al., 2014). Thus, this experimental paradigm can be considered sensitive when assessing hippocampus-based processes.

As formulated in the hypothesis section, we expect a compromised hippocampal-related acquisition of spatial context as measured by spatial discrimination ability during states high in arousal. Impairments in spatial context processing might result from an arousal-induced over-reliance on the introduced stimulus response strategy as provided by the instructed and trained task representations. Evidence supporting this prediction would be impaired performance in the spatial mnemonic discrimination paradigm in both increased aversive and appetitive arousal states as compared to the control condition.

### Materials and Methods

#### Design and Procedure

In a 3 (arousal state) × 5 (lure-type) factorial design, each participant was randomly allocated to one of the three conditions (control, violence, erotica; between-subject variable) and performed the spatial discrimination paradigm consisting of an implicit learning and a surprise recall phase with correct spatial positions and five lure displacements (1-Move, 2-Move, 3-Move, 4-Move, Corner-Move; within-subject variable). All tests took place between 10 am and 12 am.

The experimental task was developed using E-Prime software (Version 2.0; Psychology Software Tools, Pittsburgh, PA, USA; Schneider et al., 2012) and presented on a Dell 22 Monitor P2217H monitor (resolution 1920 pixels × 1080 pixels, refresh rate = 60 Hz).

#### Participants

All participants were healthy volunteers recruited from the University of Innsbruck und met the same criteria as the sample for validation of the arousal induction method. Sixty-six participants (40 females, 26 males; *M*_age_ = 22.23 years, *SD* = 2.61; age range: 18–33 years) were tested and informed consent was obtained according to the guidelines of the Ethics Committee of the Department of Psychology, University of Innsbruck.

#### Experimental Manipulation of the Arousal State

The arousal induction method was applied to the subjects of whom each was randomly allocated to one of the three arousal conditions (control, violence, erotica; *n* = 22 per condition).

#### Spatial Mnemonic Discrimination Task

The Spatial Mnemonic Discrimination Task (Reagh et al., 2014; Marshall et al., 2016) consists of 140 common objects and is structured in two sequences, an encoding and a retrieval phase. At first, participants had to judge whether each presented item is more likely to be used indoors or outdoors by responding with their right or left index finger. The objects were located in a 5 × 7 grid due to the dimensions of common widescreen displays and appeared for 2500 ms, assigned pseudo randomly (Marshall et al., 2016). This first sequence conduces incidental learning (Reagh et al., 2014).

After a 5 min delay, in the second sequence participants judged whether the same objects presented in the first sequence were located in the same or a different location. In this setting, 40 objects were placed in a repeated grid space and 100 objects had been moved. The moved objects can be divided in five different lure-types containing 20 objects each. The objects were categorized in 1-Move, 2-Move, 3-Move or 4-Move dimensions in horizontal and vertical direction and as a fifth category Corner-Move Lures, which are objects relocated to the opposite corner of the grid. Diagonal displacements were excluded in the current setting (Reagh et al., 2014). This differentiation enables one to make parametric comparisons across levels of mnemonic interference (Marshall et al., 2016). Each space of the grid is equally likely to contain an object and direction of displacements also appeared for 2500 ms assigned pseudo randomly for each participant. The task was programmed using E-Prime 2.0 (Schneider et al., 2012).

### Data Analysis

Change detection performance was quantified using *d*-prime (*d*’) as a measure of sensitivity according to signal detection theory (Macmillan and Creelman, 1991). Based on the z-transformed probability of correct match responses (hits, *H*) and incorrect match responses (false alarms, *F*) for each displacement step and condition, we calculated sensitivity separately: *d*’ = z(*H*) – z(*F*). Corrections for extreme values in hit rates or false alarms were applied following the log-linear approach (Snodgrass and Corwin, 1988; Stanislaw and Todorov, 1999) by adding 0.5 to both the number of hits and the number of false alarms and adding 1 to both the number of signal trials and the number of noise trials. The log-linear approach results in less biased estimates of sensitivity *d*’ than does the 1/(2N) rule (Hautus, 1995).

To examine the effects of different arousal states on spatial learning performance, a 3 × 5 mixed-measures ANOVA was applied to the estimates of sensitivity *d*’ with arousal state (control, erotica, violence) as between-subject variable and lure-types (1-Move, 2-Move, 3-Move, 4-Move, Corner-Move) as within-subject variable. Planned contrasts were used to decompose significant effects of arousal states on spatial discrimination ability for different lure-types. Sphericity was tested using Mauchly’s test and in case of deviance from sphericity Type I error was controlled by adjusting the degrees of freedom using the Greenhouse-Geisser correction. An alpha-level of 0.05 was used for all statistical tests. All reported *p* values are two-tailed. We determined Bayes factors for each hypothesis, which allows quantifying the relative strength of evidence for one hypothesis compared to the other. Data were analyzed using SPSS (Version 24) and JASP (Version 0.8; JASP Team 2016), respectively.

### Results

Data from all participants were used for statistical analysis. Effect sizes are reported by partial eta squared $\eta_{\text{Part}}^{2}$ (0.01 = small; 0.06 = medium; 0.14 = large).

#### Effects of Arousal States on Spatial Discrimination

**Figure 1:**
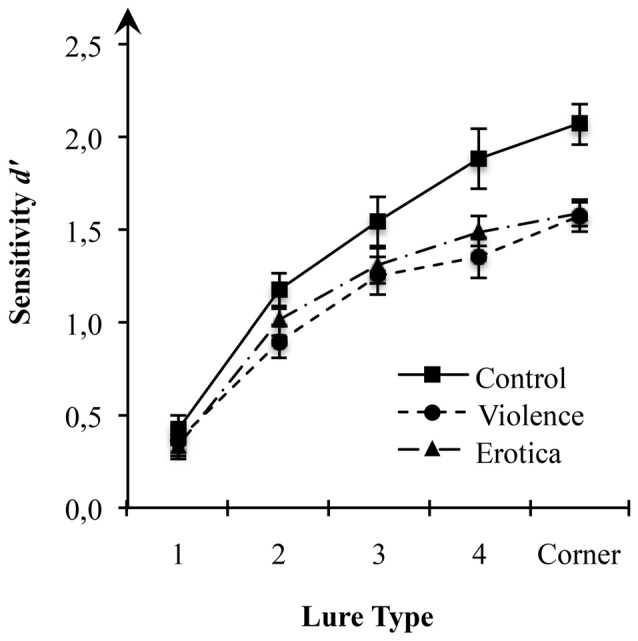
Effects of alterations in arousal on spatial discrimination performance. Compared to the control condition, both high arousal groups showed reduced sensitivity of spatial displacement as measured by d-prime (*d*’) for spatial locations at a moderate to low level of mnemonic interference. Standard errors are represented by the error bars attached to each column in the figure.

Considering the effect of arousal states on acquisition of spatial information as predicted in our hypothesis, we first performed a 3 (arousal states) × 5 (lure-types) mixed-measures ANOVA on estimates of sensitivity *d*’ for spatial locations with arousal state (control, violence, erotica) as between-subject variable and lure-types (1-Move, 2-Move, 3-Move, 4-Move, Corner-Move) as within-subject variable (Figure 1). Results showed a strong main effect for the between-subject variable arousal state, *F*_(2,63)_ = 5.13, *MSE* = 0.133, *p* = 0.009, $\eta_{\text{Part}}^{2}$ = 0.14, *BF*_10_ = 2.53. For all lure-types, planned contrasts revealed better performance in the control group (*M*_Control_ = 1.42, *SE*_Control_ = 0.10) as compared to both the aversive arousal group (*M*_Violence_ = 1.09, *SE*_Violence_ = 0.08) with a difference of 0.33 (*SE* = 0.11), Bonferroni-adjusted *p* = 0.012, as well as the appetitive arousal group (*M*_Erotica_ = 1.14, *SE*_Erotica_ = 0.05) with a difference of 0.27 (*SE* = 0.11), Bonferroni-adjusted *p* = 0.048. By contrast, there was no difference in spatial discrimination scores between the high arousal groups, Δ = 0.06, *SE* = 0.11, Bonferroni-adjusted *p* = 1.

This result pattern indicates strong impairment of spatial context processing due to the experimental arousal elicitation. Interestingly, since the latter result rules out valence specific alterations in estimates of sensitivity *d*’ for spatial information, impairments in spatial discrimination are attributable to experimental variations in arousal, regardless of the appetitive or aversive direction.

#### Interaction of Arousal States and Lure Displacements

Assessing the interaction between arousal states and lure displacement, the 3 × 5 mixed-measures ANOVA revealed a moderate interaction between the two factors arousal states and lure-types on estimates of sensitivity *d*’ (see Table 2), *F*_(8,252)_ = 2.21, *MSE* = 0.111, *p* = 0.028, $\eta_{\text{P}}^{2}$ = 0.07, main effects model: *BF*_10_ = 7.44e+68; full effects model: *BF*_10_ = 9.23e+68, adding the interaction increases the degree of this support by 1.80. Mauchly’s test indicated that the assumption of sphericity had been fulfilled for the within-subject variable lure-types $\chi_{\left(9\right)}^{2}$ = 13.59, *p* = 0.138. To decompose this statistically significant interaction between arousal states and sensitivity for lure displacement, we applied contrasts defining the lure-type with the highest level of mnemonic interference, i.e., 1-Move lures, as reference level. Planned contrasts comparing the 1-Move lures with the other levels of the factor lure-type revealed strong effects of arousal states for Corner-Move lures, *F*_(2,63)_ = 5.52, *MSE* = 0.226, *p* = 0.006, $\eta_{\text{P}}^{2}$ = 0.15, as well as moderate effects for 4-Move lures, *F*_(2,63)_ = 4.84, *MSE* = 0.252, *p* = 0.011, $\eta_{\text{P}}^{2}$ = 0.13, but not for 3-Move lures, *F*_(2,63)_ = 1.04, *MSE* = 0.271, *p* = 0.359, or for 2-Move lures, *F*_(2,63)_ = 1.67, *MSE* = 0.141, *p* = 0.197. The means reveal contrast effects reflecting altered spatial discrimination ability for high arousal states (violence, erotica) when compared to the low arousal condition (control). The lack of effects of arousal states on recognition performance in lure-types near the correct spatial position is interpreted as a result of item difficulty being too high, leading to floor effects. Lure displacements with levels of mnemonic interference too high are insensitive to effects of arousal states on spatial discrimination.

**Table 2:** Effects of alterations in arousal on spatial discrimination performance: estimates of sensitivity *d*-prime (*d*’) for spatial locations in the Spatial Mnemonic Discrimination Paradigm (Reagh et al., 2014; Marshall et al., 2016) for each lure displacement and arousal state.

	Arousal state					
	Control		Violence		Erotica	
Lure-Type	*M*	*(SE)*	*M*	*(SE)*	*M*	*(SE)*
1-Move	0.42	(0.08)	0.36	(0.08)	0.33	(0.07)
2-Move	1.17	(0.10)	0.90	(0.09)	1.01	(0.08)
3-Move	1.54	(0.14)	1.25	(0.10)	1.31	(0.10)
4-Move	1.88	(0.16)	1.35	(0.11)	1.49	(0.08)
Corner-Move	2.07	(0.11)	1.57	(0.08)	1.59	(0.07)

*Standard errors in parentheses*.

In fact, the main effect for lure-type, *F*_(4,252)_ = 176.63, *MSE* = 0.111, *p* < 0.001, $\eta_{\text{P}}^{2}$ = 0.74, *BF*_10_ = 1.31e+68, shows that increasing spatial proximity of the lure to the correct original position leads to decreased discrimination performance. This finding is in line with previous results obtained by using this paradigm to assess age-related declines in spatial discrimination ability, showing that differences between young and old participants appear in 3-Move lures and 4-Move lures, thus in the intermediate interference range (Reagh et al., 2014; Marshall et al., 2016). The authors interpreted the absence of differences in 1-Move lures and 2-Move lures, as well as Corner-Move lures, as a result of mnemonic interference being too high in the first two and too low in the latter.

#### Effects of Arousal State for Each Lure Displacement

To further decompose significant effects of arousal states on spatial discrimination performance, planned contrasts were performed comparing estimates of sensitivity *d*’ for spatial locations between all three arousal states.

One-way ANOVA with the between-subject variable arousal state on performance scores for 4-Move lures showed strongly altered spatial discrimination depending on arousal state, *F*_(2,63)_ = 5.24, *MSE* = 0.317, *p* = 0.008, $\eta_{\text{Part}}^{2}$ = 0.14, *BF*_10_ = 5.93. Similar to the contrasts decomposing the main effects of arousal states, we found better performance in the control group when compared to the aversive arousal group with a difference of 0.53 (*SE* = 0.17), Bonferroni-adjusted *p* = 0.009. Likewise, the appetitive arousal group showed slightly impaired spatial discrimination performance than the control group with a difference of 0.39 (*SE* = 0.17), Bonferroni-adjusted *p* = 0.072, but their sensitivity *d’* did not differ from the aversive arousal group, with a difference of 0.14 (*SE* = 0.17), Bonferroni-adjusted *p* = 1.

Next, we examined the effect of arousal states on spatial discrimination for Corner-Move lures via one-way variance analysis on estimates of sensitivity *d*’. We found a strong alteration of performance scores between arousal groups, *F*_(2,63)_ = 9.69, *MSE* = 0.182, *p* < 0.001, $\eta_{\text{Part}}^{2}$ = 0.24, *BF*_10_ = 128.20. In line with our prediction, both the aversive arousal group and the appetitive arousal group showed impaired spatial discrimination compared to the control group. The former with a difference of −0.50 (*SE* = 0.13), Bonferroni-adjusted *p* < 0.001, and the latter with a difference of −0.48 (*SE* = 0.13), Bonferroni-adjusted *p* < 0.001. Again, the difference in performance scores of 0.02 (*SE* = 0.13) between the high arousal states did not reach significant levels, Bonferroni-adjusted *p* = 1.

By contrast, as indicated by planned contrasts, one-way ANOVAs confirmed that neither spatial discrimination for 1-Move, *F*_(2,63)_ = 0.38, *MSE* = 0.140, *p* = 0.685, *BF*_10_ = 0.17, nor for 2-Move, *F*_(2,63)_ = 2.18, *MSE* = 0.182, *p* = 0.122, *BF*_10_ = 0.64, or for 3-Move lures, *F*_(2,63)_ = 0.288, *MSE* = 0.52, *p* = 0.173, *BF*_10_ = 0.48, were affected by arousal states.

In line with our previous statistical analysis, results further suggest that estimates of sensitivity *d’* for spatial locations of the three groups begins to diverge at a moderate level of mnemonic interference (4-Move lures) and reaches its strongest alteration at a low level of mnemonic interference (Corner-Move lures). Once again, our results uniformly show impaired spatial discrimination in high arousal states relative to a neutral state.

Compared to the control condition, both high arousal groups showed strong impairment of spatial context acquisition. Presumably due to floor effects, alterations in performance reached significance at a moderate to low level of mnemonic interference. Our behavioral data support the idea that states high in arousal disrupt hippocampus-dependent acquisition of spatial context information (Schwabe et al., 2007; Brunyé et al., 2017; Vogel et al., 2017) as indicated by impaired spatial discrimination ability (Reagh et al., 2014; Marshall et al., 2016). Consistent with our second hypothesis, high arousal states did not differ in the direction and magnitude they hamper performance, thus excluding a role of their valence, i.e., appetitive or aversive (Mather and Sutherland, 2009; Harmon-Jones et al., 2013).

## Experiment 2: Arousal and Sequence Acquisition

The purpose of Experiment 2 was to explore how arousal states affect acquisition of sequence information. Sequence information refers to a type of context which informs actual behavior about the chronological order of events, thus allowing to predict a relevant event by preceding cues. In contrast to the formation of simple stimulus–response (S–R) associations underlying habit formation (Hull, 1943), prediction learning requires acquisition of stimulus–stimulus (S–S) relationships (Tolman, 1948), therefore the two processes might rely on different memory systems (Bornstein and Daw, 2012; Mattfeld and Stark, 2015). Both neuroimaging (Ross et al., 2009) and neuropsychological evidence (Schapiro et al., 2014) support the role of hippocampus in formation of sequential associations, presumably based on its function in predicting the next event in a sequence (Turk-Browne et al., 2010; Moustafa et al., 2013; Davachi and DuBrow, 2015). The hippocampus is assumed to promote binding of temporally close events, thus embedding incoming information in a temporal context (Staresina and Davachi, 2009; Hsieh et al., 2014).

Previous findings regarding stress effects on associative learning using eye-blink conditioning paradigms have proved equivocal. Whereas learning performance in a delay and trace eye-blink conditioning paradigm requiring the acquisition of simple relations is enhanced under acute arousal (Duncko et al., 2007), providing additional context using eye-blink conditional discrimination learning conditioning is strongly impaired in aroused humans (Wolf et al., 2012). Instead of simply learning CS-US pairings, the latter paradigm requires acquisition of a contextual cue in terms of a preceding discriminative stimulus, which indicates the reliability of the CS to predict the US, a process related to hippocampal function (Green and Woodruff-Pak, 2000; McDonald et al., 2002; MacDonald, 2008).

Based on the findings that arousal hampers the engagement of memory systems supporting context acquisition (McDonald et al., 2007; Packard and Goodman, 2012; Schwabe and Wolf, 2013), we hypothesize disrupted acquisition of sequential context by increased arousal states. To test our prediction, human subjects underwent the validated arousal induction procedure to experimentally modulate arousal state. Thereafter, participants performed an associative learning paradigm, the learned irrelevance task (Orosz et al., 2008), which requires continuous acquisition of sequences to predict a target cue. The capability to acquire sequential context was inferred from their ability or inability, respectively, to accelerate responses to the behavioral relevant cue using reliable predictor cues. Slower target responses after reliable cues due to increases in arousal would indicate disrupted context acquisition, providing evidence for our main hypothesis. In addition, the absence of differences in performance during both states high in arousal would suggest that alterations in arousal could be considered pivotal, regardless of their motivational direction.

### Materials and Methods

#### Design and Procedure

We designed a 3 (arousal state) × 3 (predictive association) factorial experiment. Participants were randomly allocated to one of the three conditions (control, erotica, violence; between-subject variable) and performed the learned irrelevance paradigm consisting of an implicit learning and a recall phase. Participants were told they would perform a simple response speed testing task, during which they had to respond as fast as possible to a target letter. However, each target letter was predicted by three types of conditioned stimuli (random, pre-exposed (PE), non pre-exposed (NPE); within-subject variable) which differed in their predictive reliability. Like in Experiment 1, all tests took place between 10 am and 12 am.

The experimental task was developed using E-Prime software (Version 2.0; Psychology Software Tools, Pittsburgh, PA, USA; Schneider et al., 2012) and presented on a Dell 22 Monitor P2217H monitor (resolution 1920 pixels × 1080 pixels, refresh rate = 60 Hz).

#### Participants

Eighty-four healthy volunteers (51 females, 33 males; *M*_age_ = 22.85 years, *SD* = 2.36; age range: 19–34 years) recruited from the University of Innsbruck were tested. All participants met the same inclusion criteria as the sample for validation of the arousal induction method. Informed consent was obtained according to the guidelines of the local Ethics Committee.

#### Experimental Manipulation of the Arousal State

Participants underwent the arousal induction method, each one being randomly allocated to one of the three arousal conditions (control, violence, erotica; *n* = 28 per group).

#### Learned Irrelevance Paradigm

The Learned Irrelevance Paradigm was designed as a visual target detection task (Orosz et al., 2008, 2011). Participants saw a series of letters in equal size and white color in the middle of a black screen and were instructed to respond as fast as possible to the target letter X immediately when it occurred by pressing the space bar on the keyboard. There were 450 letters (75 target and 375 non-target), each presented for one second one after another without interstimulus interval, resulting in an overall test duration of 7.5 min.

In addition to the target letter “X”, 10 additional letters were presented. These non-target characters were divided into two groups, the PE character group consisting of a selection of five consonants (B, D, T, Y and Z) and the NPE character group consisting of five vowels (A, E, I, O and U). The predictability of the target by the pre-target letter was manipulated in three steps as described in the following sequence type. First, in the random condition (R) each letter from the PE group randomly appeared between the target letters. Hence, the pre-target letter did not predict the target letter. Second, in each block of the NPE condition the target letter was predicted five consecutive times by the same letter from the NPE group, therefore representing a CS for the occurrence of the target letter. Since one NPE character was used as CS only one single time in the whole task and was never presented elsewhere, NPE-CS should have a high predictive value and associability. By contrast, in the third and other PE condition the target was predicted five consecutive times by just one particular PE-letter and similar to the NPE condition, each character was used for one block only. The crucial distinction was that unlike letters from the NPE group, PE letters were presented as filler letters in each preceding block. Since PE-CS occurred both as fillers and pre-target letters, they constituted less reliable predictors of the target letter and it should be harder to learn an association between PE-CS and the target.

Each condition was segmented in five blocks containing 30 characters with five targets, five target predictors and 20 fillers, resulting in 15 blocks overall. The number of filler letters between target and following predictor varied from one to eight with an average of four. Filler characters were letters from the PE group. Blocks were presented in a fixed order, always beginning with R and subsequently being counterbalanced with either PE or NPE. Two successive blocks never belonged to the same condition.

According to the degree of prediction, the latency measured between the target onset and key pressing was expected to be the lowest in NPE, somewhat higher in PE and highest in R. Faster responses in PE- and NPE-blocks compared to R-blocks would indicate that participants have learned the associative sequences, i.e., the predictive letter-letter associations. In addition, the average reaction time (RT) in PE is expected to be significantly higher than in NPE. This would indicate that participants had learned from previous trials involving PE characters that this group of letters does not predict the target letter. Therefore, differences in RT in the NPE- and the PE-condition represent a measure of the learned irrelevance effect (Orosz et al., 2008).

### Data Analysis

Supposing that the first two predictor-target pairings generated a predictive association enabling anticipations of the following target sequences, only the last three target RTs were utilized for the analysis (Orosz et al., 2011). The mean RT was operationalized as dependent variable, whereby differences between trial types around and below zero indicated disrupted associative learning (Orosz et al., 2008).

As opposed to the analysis strategy in Experiment 1, where comparisons between groups within each lure displacement were crucial to test our hypothesis, this time we compared response latencies between sequence types within each arousal state. Since alterations in response times indicate whether stable associations between within predictor-target pairings were built, the absence of accelerated responses after a predictor suggests impaired acquisition of predictive sequences whereas faster responses prove successful learning of context information. First, we applied a 3 × 3 mixed ANOVA with the between-subject factor arousal state (control, violence, erotica) and the within-subject factor sequence type (R, PE, NPE). Second, in order to decompose interaction effects, we calculated planned contrasts in terms of additional repeated measures ANOVAs for each factor level of the between-subject variable arousal state.

All reported *p* values are two-tailed and alpha levels were set at 0.05. Again, we applied Bayesian inferential procedures for each hypothesis testing. Data were analyzed using SPSS (Version 24) and JASP (Version 0.8; JASP Team 2016).

### Results

Response times for correct responses from all participants were used for data analysis. To deal with outliers, we applied the median absolute deviation method (Leys et al., 2013) to response times for each condition of each factor separately. In doing so, a total of 3.62% of all trials were identified as outliers and therefore eliminated. Data from all tested participants were included for data analysis.

#### Interaction of Arousal States and Sequential Stimulus–Stimulus Pairings

**Figure 2:**
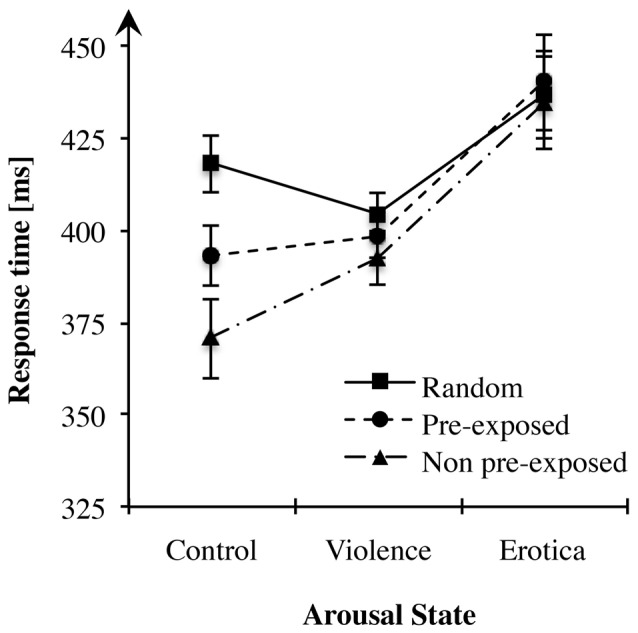
Effects of alterations in arousal on associative learning performance. In both high arousal conditions response times did not differ between sequence types, whereas in the control group participants showed fastest responses in non pre-exposed (NPE)-trials, slowest responses in R-trials with pre-exposed (PE)-trials lying in between, thus learning performance remained unchanged. Standard errors are represented by the error bars attached to each data point in the figure.

The effects of alterations in arousal on behavioral responses (see Table 3 and Figure 2) was analyzed using a 3 (arousal state) × 3 (sequence type) mixed measures ANOVA with different groups (control, violence, erotica) as between-subject variable and types of *sequences* (random, PE, NPE) as within-subject variable. Mauchly’s test indicated that the assumption of sphericity had been violated for the within-subject variable $\chi_{\left(2\right)}^{2}$ = 9.76, *p* = 0.008. The degrees of freedom were corrected using Greenhouse-Geisser estimates of sphericity (ε = 0.90). We found a strong interaction between both factors, *F*_(3.59,145.31)_ = 9.44, *MSE* = 478.641, *p* < 0.001, $\eta_{\text{P}}^{2}$ = 0.19, main effects model: *BF*_10_ = 1.43e+6; full effects model: *BF*_10_ = 2.85e+10. In line with our prediction, means revealed effects reflecting altered prediction learning for high arousal states in comparison to the low arousal condition. In addition, results revealed a main effect for CS-type, *F*_(1.79,145.31)_ = 20.72, *MSE* = 478.641, *p* < 0.001, $\eta_{\text{P}}^{2}$ = 0.20, *BF*_10_ = 77, 025.39. To further decompose both the interaction of arousal states and sequence type as well as the main effect of sequence type, in the next paragraph we applied one-way ANOVAs on sequential stimulus–stimulus learning performance for each arousal group individually.

**Table 3:** Effects of alterations in arousal on associative learning performance: response times in the Learned Irrelevance Paradigm (Orosz et al., 2008) for each trial type and arousal state.

	Arousal state					
	Control		Violence		Erotica	
Sequence type	*M*	*(SE)*	*M*	*(SE)*	*M*	*(SE)*
Random	418.08	(7.77)	404.48	(5.70)	436.60	(11.76)
Pre-exposed	393.08	(8.19)	398.60	(6.05)	440.11	(13.26)
Non pre-exposed	370.68	(10.88)	392.36	(7.11)	434.51	(12.43)

*Standard errors in parentheses*.

Considering the effect of alterations in arousal on overall response times, the 3 × 3 ANOVA showed a strong main effect for the factor arousal state, *F*_(2,81)_ = 6.81, *MSE* = 2, 308.874, *p* = 0.002, $\eta_{\text{P}}^{2}$ = 0.14, *BF*_10_ = 19.30. The appetitive arousal group (*M*_Erotica_ = 437.07 ms, *SE*_Erotica_ = 12.23) responded more slowly to the target letters compared to the control group (*M*_Control_ = 393.95 ms, *SE*_Control_ = 8.34) with a difference of 43.13 ms (*SE* = 12.84), Bonferroni-adjusted *p* = 0.003. Likewise, compared to the aversive arousal group (*M*_Violence_ = 398.48 ms, *SE*_Violence_ = 5.31), RTs in the appetitive arousal condition slowed down with a difference of 38.59 ms (*SE* = 12.84), Bonferroni-adjusted *p* = 0.012. By contrast, the difference of 4.54 ms (*SE* = 12.84) between the control and the negative arousal conditions was not significant, Bonferroni-adjusted *p* = 1. Although exposure to aversive content had no effect on response times, an increase in appetitive arousal led to adeceleration of behavioral responses.

#### Effects of Arousal State for Each Sequence Type

To assess whether increases in arousal change the acquisition of stimulus–stimulus sequences, we tested for discrepancies in response times for different predictive associations for all three arousal states separately.

First, we tested learning performance operationalized as alterations in response times between sequence types in the control condition. As Mauchly’s test indicated that the assumption of sphericity had been violated $\chi_{\left(2\right)}^{2}$ = 7.46, *p* = 0.024, degrees of freedom were corrected using Greenhouse-Geisser estimates of sphericity (ε = 0.80). Repeated measures ANOVA produced a strong main effect for the within-subject variable sequence type, *F*_(1.60,43.22)_ = 30.15, *MSE* = 652.633, *p* < 0.001, $\eta_{\text{Part}}^{2}$ = 0.53, *BF*_10_ = 5.01e+6. More precisely, contrasts revealed that participants achieved faster responses after both the PE, *F*_(1,27)_ = 23.77, *MSE* = 736.465, *p* < 0.001, $\eta_{\text{Part}}^{2}$ = 0.47, and the NPE predictors, *F*_(1,27)_ = 40.24, *MSE* = 1, 563.369, *p* < 0.001, $\eta_{\text{Part}}^{2}$ = 0.60, than after random stimuli. Likewise, faster reactions were measured for target letters predicted by NPE compared to PE stimuli, *F*_(1,27)_ = 16.85, *MSE* = 833.912, *p* < 0.001, $\eta_{\text{Part}}^{2}$ = 0.38. These results are in accordance with previous studies, clearly replicating the learning effects in the learned irrelevance paradigm within the control *condition* (Orosz et al., 2008).

Second, we applied a repeated measures ANOVA on learning performance of the aversive arousal group. Mauchly’s test indicated that the assumption of sphericity had been violated $\chi_{\left(2\right)}^{2}$ = 6.21, *p* = 0.045. Again, degrees of freedom were corrected using Greenhouse-Geisser estimates of sphericity (ε = 0.83). Results showed no effect of sequence type on RTs, *F*_(1.65,44.54)_ = 2.09, *MSE* = 596.042, *p* = 0.143, *BF*_10_ = 0.52, indicating that participants did not anticipate the target using the prediction stimuli. Hence, acquisition of sequential context was abolished within the aversive arousal condition.

Next, we addressed differences between predictive stimulus-stimulus associations within the appetitive arousal condition. Like for the aversive arousal group, repeated measures ANOVA indicated no alterations in response times depending on the levels of the experimental factor sequence type, *F*_(2,54)_ = 0.82, *MSE* = 274.098, *p* = 0.446, *BF*_10_ = 0.20. Mauchly’s test indicated that the assumption of sphericity had been fulfilled for the within-subject variable lure-types $\chi_{\left(2\right)}^{2}$ = 0.93, *p* = 0.627. Thus, appetitive arousal abolished acquisition of sequence information and thereby anticipation of the required response.

The general objective of Experiment 2 was to examine effects of high arousal states on sequential context processing. To summarize, in highly aroused subjects response times did not differ between sequence types, indicating abolished learning of simple predictive pairings, whereas in the control group, learning performance was fully intact and adapted to the experimentally manipulated predictive value of the pre-target stimulus. This result pattern supports our prediction stating disrupted acquisition of sequential context by states of increased arousal associated with a hippocampus related, “cognitive” system (Rajah et al., 2010a; Vogel et al., 2015a). Intriguingly, high arousal states modulated context processing regardless of their motivational direction. By contrast, in a control state not manipulated, human subjects learned stimulus-stimulus associations and used this sequence information to predict the behaviorally relevant target as indicated by accelerated response times.

## Discussion

We actively represent ongoing events within a spatial-temporal context, which informs behavior to respond adaptively to current demands. In everyday life, binding spatial-temporal contextual details from the incoming flow of information to a current event mainly occurs in an implicit way. The goal of the present experiments was two-fold: first, we aimed to examine how variations in arousal impact two kinds of context processing, the acquisition of spatial (Experiment 1) and sequential (Experiment 2) associations. Second, we intended to test whether arousal states of different motivational direction, i.e., aversive and appetitive valence, promote different effects on context acquisition. Our findings show that regardless of valence, increased arousal interferes with implicit learning of contextual information to support task performance. Both aversively and appetitively aroused human subjects showed impaired ability to acquire spatial relations in a spatial discrimination paradigm (Experiment 1) and failed to detect predictive sequences in an associative learning paradigm (Experiment 2). Thus, under increased arousal, participants failed to bind or use spatial and sequential context to inform behavioral responses and thereby facilitate performance. States high in arousal might favor reflexive action by narrowing the attentional scope on executing implemented stimulus-response bindings (Packard and Goodman, 2012; Schwabe and Wolf, 2013; Gagnon and Wagner, 2016; Schwabe, 2016). As a consequence, sensitivity for spatial-temporal contextual details is reduced, promoting impaired context processing. We conclude that sensitivity for spatial-temporal context in terms of implicit acquisition of spatial and sequential associations varies as a function of arousal state.

How can this variation in the use of contextual cues altered by arousal be explained? One line of research which offers an explanation showed decreased engagement of hippocampal and presumably prefrontal cortical systems under stress (Schwabe and Wolf, 2013). Neuropsychological evidence supports the role of hippocampus in constructing spatially coherent internal scenes and an inflexible focus on specific fragments in patients with selective bilateral hippocampal damage (McCormick et al., 2017). In addition, at least in young adults, changes in the ability to retrieve both spatial and temporal contextual details are related to inter-individual differences in hippocampal volumes (Rajah et al., 2010a). In concert with other brain sites, the interconnected hippocampus (Ranganath and Ritchey, 2012; Yonelinas, 2013) supports binding of spatial-temporal contextual details with item information while required to integrate actual experience in a contextualized episodic event (Chun and Phelps, 1999; Hannula and Ranganath, 2008; Shimamura and Wickens, 2009; Zeidman and Maguire, 2016). Thus, weakening the engagement of hippocampus-centered learning strategies may hamper the processing of spatial-temporal context. Increased arousal is one influential factor among others (Packard and Goodman, 2013) that bias the competition of active learning strategies towards a dominance of striatum-dependent, habit-like stimulus-response learning and weakens hippocampus-dependent, cognitive learning (Packard and Goodman, 2012; Schwabe and Wolf, 2013; Schwabe, 2016). In the light of our findings, impaired performance by states high in arousal results from a reduced use of spatial-temporal contextual cues, which might reflect weakened engagement of a hippocampus-centered “cognitive strategy” (Vogel et al., 2015a,b, 2017) leading to a measurable behavioral impairment in task performance. Nevertheless, it is noteworthy that both experiments were of behavioral nature, therefore assumptions about the involved brain areas remain hypothetical.

Arousal and underlying locus correuleus-norepinephrine activity orchestrate cognition in several ways (Berridge and Waterhouse, 2003) by promoting exploitation of active behavioral sets (Aston-Jones and Cohen, 2005) as well as reorienting (Bouret and Sara, 2005; Sara and Bouret, 2012; Sara, 2015) and narrowing attention to high-priority information in memory and cognition (Mather and Sutherland, 2011; Harmon-Jones et al., 2013; Mather et al., 2016). These consequences of increased arousal for cognition might be reflected by our findings. In our study, states high in arousal might have reoriented attention to the active stimulus-response set, which has been established by task instructions and completion of practice trials, thus narrowing active cognition to the behaviorally relevant cues (i.e., indoor or outdoor objects in Experiment 1, target letter in Experiment 2) at the cost of implicit sensitivity to their spatial or temporal context.

In the accounts mentioned, arousal-biased cognition is assumed to exert an adaptive value by promoting a less resource-demanding mode as reflected by enhanced reliance on stimulus-response learning and habitual responding, as well as reduced engagement of more complex cognitive strategies (Packard and Goodman, 2012; Schwabe and Wolf, 2013). As demonstrated by our experiments, these changes in information processing lead to impaired implicit acquisition of spatial and sequential cues at the same time, resulting in performance decrements in tasks which require context processing to facilitate task execution. But how can reduced sensitivity for spatial-temporal contextual details be an adaptive response in challenging situations? One reasonable suggestion comes from more recent research in the domain of perceptual decision-making. Krishnamurthy et al. (2017) provided evidence by showing that arousal adaptively adjusts the influence of prior expectations on perceptual judgments. In their study, increased arousal in highly dynamic environments promoted less influence of priors on the perception of a stimulus. Similarly, in our study participants were exposed to highly arousing events which might have signaled an uncontrollable situation with unpredictable outcomes. Thus, reduced use of contextual details to inform behavioral responses might be adaptive in unpredictable environments, since information on spatial positions or sequential orders of events may change quickly and therefore represent unreliable information.

Our results are well in line with previous findings, showing impaired spatial cognition (e.g., Olver et al., 2015) and associative learning (e.g., Wolf et al., 2012) by stress. The present findings, however, extend previous evidence in several important ways. First and foremost, most previous research focused on stress-induced impairments in behavioral tasks directly addressing the instructed and therefore explicit remembering of contextual cues. By contrast, in the current experiments, paradigms required acquisition of spatial-temporal associations in an implicit manner. Second, we considered differences in valence of arousal states, showing that acute arousal *per se*, regardless of the motivational direction, hampers implicit context processing. Third, whereas stress effects on long-term memory are very well documented (Roozendaal et al., 2009; Joëls et al., 2011; Schwabe, 2016), our study addressed how context processing is affected *within* a state of increased arousal, i.e., immediately after a challenging encounter, which occurs in the context of the experimental task. Finally, since the presence of alterations in brain responses as measured by neurophysiological assessments can be associated with normal cognitive functioning, our experimental design allowed us to measure arousal effects on cognition on a behavioral level (Harvey, 2012).

Despite the application of reliable experimental paradigms (Orosz et al., 2008; Marshall et al., 2016) and results providing strong evidence (Lee and Wagenmakers, 2014) for the derived predictions, the present study has some limitations. One clear limitation of our study is that although we refer to neurobiological models of arousal driven modulations of cognitive processes, no neurophysiological techniques were applied. Moreover, arousal induction method was applied immediately after participants were instructed and performed the practice trials and instantly before task execution. Arousing material probably acted as distractor occupying working memory resources and thereby taxing the implicit acquisition of contextual information. Although performance decrements by arousal and distraction in concert are not exclusive, existing evidence shows that neither implicit spatial learning (Vickery et al., 2010) nor implicit sequence learning (Kaufman et al., 2010) are affected by interference from working memory load. We suggest that increased arousal impairs sensitivity to contextual details by a narrowed focus on the active stimulus-response set, at the cost of information surrounding an event. Putatively, at a neural level this shift in processing mode is reflected by decreased engagement of hippocampal- and prefrontal-centered cognitive systems supporting context processing.

To conclude, we shed light on the online processing of context information as a crucial aspect of active cognition (Davachi and DuBrow, 2015), whereas most other studies focused on the effects of states high in arousal on storing information over the long term. Our findings go beyond the known effects of stress on working memory related functions (Shields et al., 2016b), showing that increased arousal impairs implicit acquisition of spatial and sequential context of an event. Decreased sensitivity for contextual details is attributable to changes in arousal state, regardless of its motivational direction. This finding emphasizes that this bias might occur in aversive as well as appetitive conditions, such as panic and likewise in states of sexual excitement. Our study highlights the ability of alterations in general arousal to promote adjustments of ongoing cognition, thus extending our understanding of information processing during states high in arousal in everyday life and clinical populations with hyper-arousal disorders.

## Author Contributions

TM initiated and designed the study. TM, MM, MF and PS collected the data and interpreted the results. TM, BW, SZ and JP performed the preprocessing of pupil measurements. TM performed the statistical analyses and drafted the article. TM, MM, MF, BW, JP, SZ and PS read and corrected versions of the manuscript.

## Conflict of Interest Statement

The authors declare that the research was conducted in the absence of any commercial or financial relationships that could be construed as a potential conflict of interest.
